# Camellia sinensis Assisted Synthesis of Copper Oxide Nanoparticles (CuONPs) and Assessment of Its Antioxidant Activity and Zebrafish Embryonic Toxicology Evaluation

**DOI:** 10.7759/cureus.50220

**Published:** 2023-12-09

**Authors:** Aardra B S, Sandhya Sundar, Rajeshkumar Shanmugam, Ramya Ramadoss, Suganya Panneerselvam, Pratibha Ramani

**Affiliations:** 1 Oral Pathology and Microbiology, Saveetha Dental College and Hospitals, Saveetha Institute of Medical and Technical Sciences, Saveetha University, Chennai, IND; 2 Pharmacology, Saveetha Dental College and Hospitals, Saveetha Institute of Medical and Technical Sciences, Saveetha University, Chennai, IND

**Keywords:** camellia sinensis, danio rerio, copper nanoparticles, nanoparticles, copper, tea, oolong tea, embryonic toxicology, zebrafish, antioxidant

## Abstract

Background:-

*Camellia sinensis, *or oolong tea, is a partially fermented version of tea used in Asian countries. The remarkable reduction activity of the tea extract can potentially be used for synthesizing nanoparticles. Recently, *Camellia sinensis* has gained popularity for the formulation of some metal nanoparticles.

Aim

To formulate green synthesis of copper oxide nanoparticles (CuONPs) mediated by *Camellia sinensis* (oolong tea) and assess its cytotoxicity and antioxidant properties.

Materials & Methods

Oolong tea extract is prepared and added to CuSO_4_ solution to synthesize CuO nanoparticles (CuONPs). The centrifugation pellet of CuONPs is collected and subjected to DPPH (2,2 - diphenyl -1- picrylhydrazyl hydrate) and H_2_O_2_ assays. The cytotoxicity screening is performed using zebrafish embryos.

Results

The reducing activity of oolong tea successfully synthesizes the copper nanoparticles. High values are obtained in DPPH (63% inhibition at 10µL concentration, 73% inhibition at 20µL, 80% at 30µL, 85% at 40µL and 90% at 50µL concentrations) and H_2_O_2_ (50% inhibition at 10µL concentration, 65% at 20µL, 68% at 30µL, 75% at 40µL and 80% at 50µL concentrations) assays. There are no morphological deformities in the zebrafish and no loss of cell viability or delayed hatching at low concentrations (below 4-8 µL), as shown by the viable embryos with no morphological deformities.

Conclusion

The study has evidenced high antioxidant activity and minimal cytotoxicity of CuO nanoparticles produced using *Camellia sinensis*, thus proving it to be a good biomaterial for a wide range of biological applications.

## Introduction

Nanoparticles are scientifically defined as solid particles or dispersions of particles with a size that ranges from 10 to 1000 nm. Since it is easy to manipulate nanoparticles' surface characteristics and particle sizes, they can be used as very effective drug delivery systems using active and passive drug targeting. However, due to their small size and large surface area, nanoparticles are susceptible to particle-to-particle aggregation, which inhibits and hinders effective drug loading. Currently, nanoparticles have a vast plethora of applications in the field of biomedicine and not just in drug delivery. Nanoparticles are DNA carriers in gene therapy, nano biosensors, etc. [1].

Various methods are currently being employed for the synthesis of nanoparticles, including physical methods such as evaporation, condensation, and laser ablation and chemical methods such as chemical reduction, electrochemical methods, and photochemical reduction. Biosynthesis or green synthesis is the process of synthesis of a compound or chemical by natural means, such as plant extracts or animal tissues. This method is always preferred to chemosynthesis or synthesis by chemical means, as it reduces the chances of the synthesized material being cytotoxic. Biosynthesis can also help in controlling the particle morphology of the synthesized materials. Nanoparticles synthesized by green synthesis always show a better profile regarding their physical, chemical, molecular, and/or therapeutic properties [2]. Therefore, in our study, we have chosen biosynthesis or green synthesis to fabricate nanoparticles.

The DPPH (2,2 - diphenyl -1- picrylhydrazyl hydrate) assay is considered valid, accurate, easy, and economical to evaluate the radical scavenging activity of antioxidant compounds. It is currently considered the gold standard for the analysis of antioxidant activity. This is because the radical compound obtained is stable and does not need to be generated [3]. By the DPPH assay, copper oxide nanoparticles show highly efficient antimicrobial and antioxidant activity against different strains of bacteria [4,5], such as Pseudomonas aeruginosa and Escherichia coli. CuO nanoparticles have highly efficient antibacterial activity even against drug-resistant bacteria [6]. Due to these reasons, we have decided to choose the copper oxide nanoparticles for further in-depth investigation.

*Camellia sinensis*, or oolong tea, is a variety of tea commonly consumed in Asian countries such as China and Taiwan. It is partially fermented, and an eight-ounce cup contains about 10-60 mg of caffeine. Camellia sinensis contains a high amount of antioxidants such as catechins, theaflavins, and thearubigins. The phenolic extract of the tea is reported to have higher antioxidant activity than the aqueous extract [7].

Theasinensin is one of the active compounds in oolong tea, which has been shown to have high anti-inflammatory activity by reducing the levels of pro-inflammatory mediators such as interleukins and tumor necrosis factors [8,9]. Due to the presence of theasinensin, oolong tea is a potential drug for obesity and fatty liver because of its inhibitory action on pancreatic lipase activity and the enhancing effect of caffeine on noradrenaline-induced lipolysis in adipose tissue [10,11]. Oolong tea extract also has high anti-cariogenic activity [10,12] and is effective against cardiovascular diseases [13].

The high antioxidant activity possessed by *Camellia sinensis* provides an opportunity to augment potential antioxidant compounds. The study aims to analyze whether *Camellia sinensis*-mediated CuO nanoparticles have increased antioxidant activity. A favorable result opens up prospects for the employment of *Camellia sinensis* influence copper oxide nanoparticles as a better antioxidant agent than current antioxidants in commercial use.

## Materials and methods

Preparation of oolong tea extract

**Figure 1:**
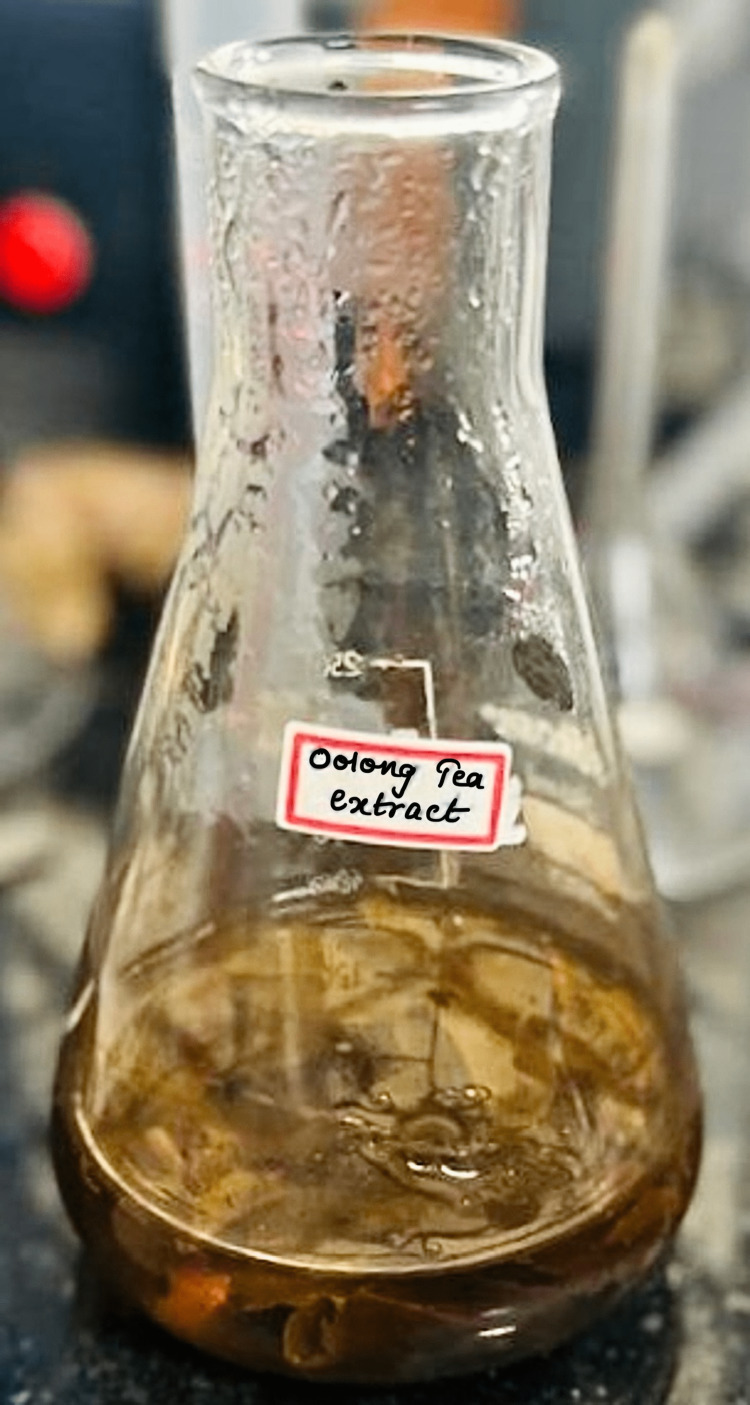
Oolong tea solution

Powdered *Camellia sinensis* leaves were purchased from SAN-CHA Tea, New Delhi, India. 10.60 g of powdered Camellia sinensis was added to 100 mL of distilled water and kept in the heating mantle for 15 - 20 mins. After it reached boiling temperature, 50 mL of the solution was filtered out to get the Camellia sinensis extract (Figure 1).

Synthesis of copper oxide nanoparticles

**Figure 2:**
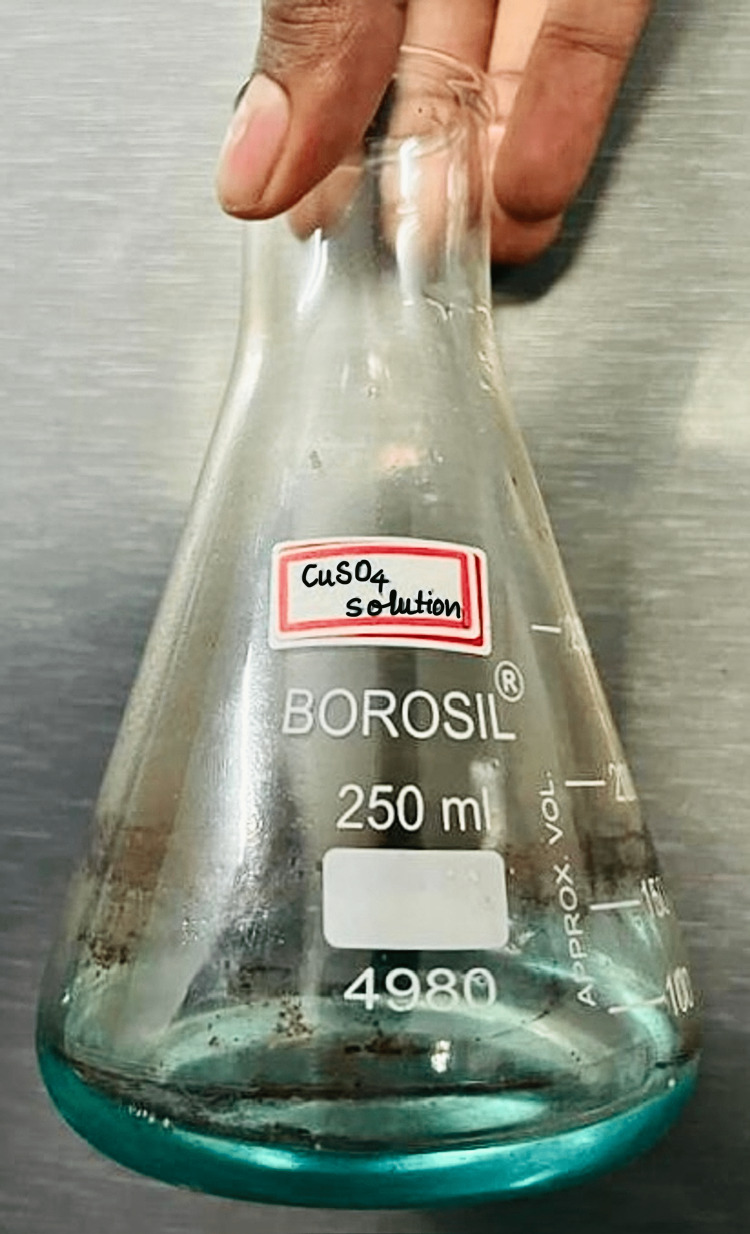
Copper sulphate solution

0.477 g of copper sulfate was added to 50 mL of distilled water to obtain the copper sulfate solution (Figure 2).

**Figure 3:**
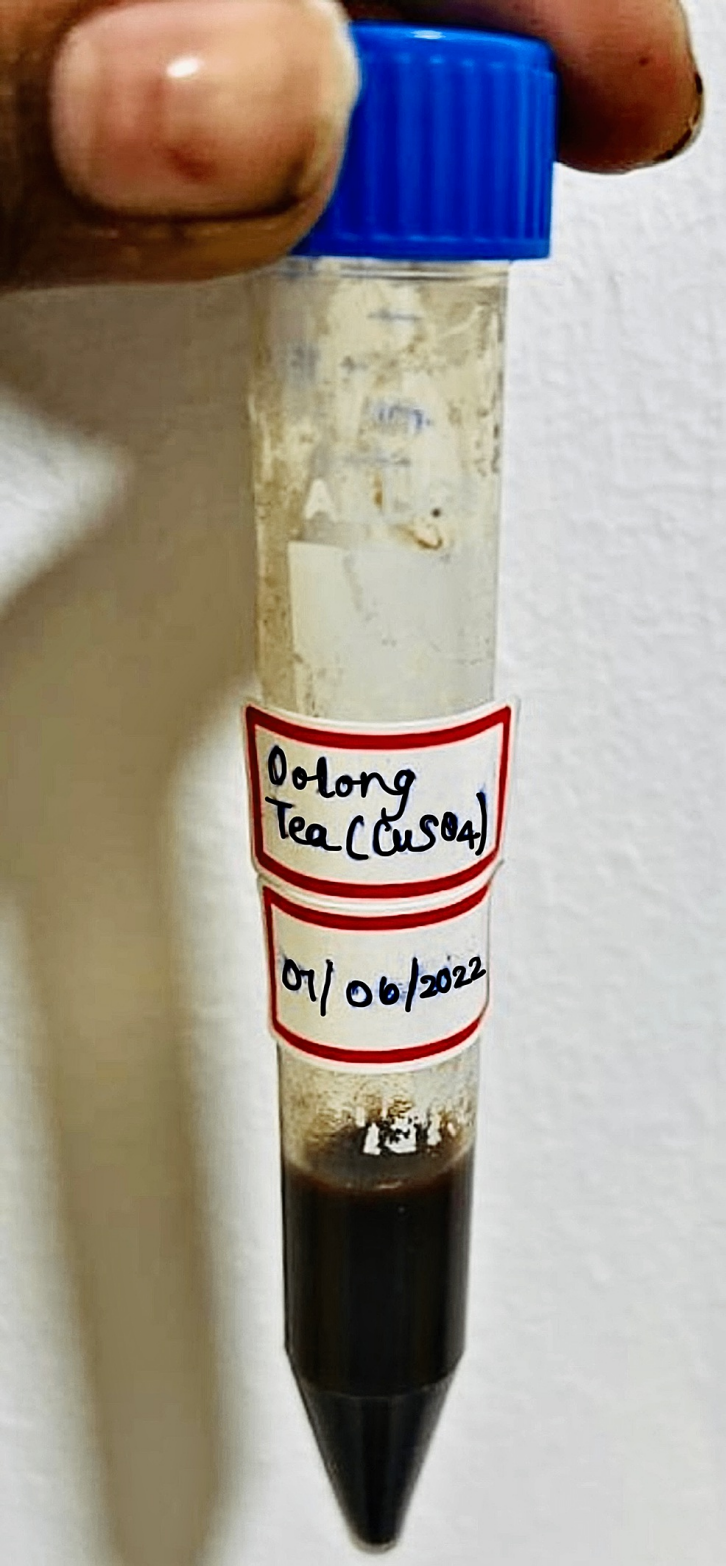
Oolong tea copper sulphate solution

The prepared 50 mL of Camellia sinensis extract was added to the 50 mL copper sulfate solution in the conical flask to obtain 100 mL of Camellia sinensis-mediated CuO nanoparticles. The mixture was covered with aluminum foil and placed in the shaker. The UV readings were taken for the Camellia sinensis-mediated CuO nanoparticle sample at specific intervals. After 48 hrs, 14 mL of the sample was kept for centrifugation in a centrifuge at 8000 rpm for 10 mins (Figure 3).

The supernatant obtained after centrifugation was discarded, and the pellet was collected using a filler. This pellet was used for further biochemical analyses.

Scanning Electron Microscopy (SEM) imaging

The nanoparticles extracted from the Camellia sinensis extract were cleaned in a reagent-grade solvent and rinsed with a reagent-grade isopropyl alcohol (IPA). The samples were placed in a nitrogen-filled, resealable container and mounted on the instrument stub. The sample was oriented to the degree that the longitudinal axis of the sample curvature was aligned with the axis of the secondary detector. The magnification was then increased, and the image was captured.

Energy Dispersive X-ray (EDX) analysis

An electron beam was scanned across the surface of the nanoparticles extracted from the Camellia sinensis extract, and the electrons were allowed to strike and stimulate the sample. Almost instantaneously, as each element returned to its original energy state, it emitted X-rays of specific energies at different wavelengths characteristic of the element. These results were plotted with X-ray wavelength on the X-axis and intensity on the Y-axis, and each corresponding element was labeled. The elements were identified by matching the X-axis's peak values with known wavelengths for each element to reveal the sample's elemental composition.

Antioxidant activity analysis by DPPH assay

Antioxidant activity was tested using the DPPH (2,2 - diphenyl -1- picrylhydrazyl hydrate) assay. Different concentrations (10 µL, 20 µL, 30 µL, 40 µL, 50 µL) of the Camellia sinensis mediated CuO nanoparticles were mixed with 1 mL of 0.1 mM DPPH in methanol and 450 µL of 50 mM Tris-HCl buffer (pH 7.4) and incubated for 30 mins. The reduction in the quantity of DPPH free radicals was assessed dependent on the absorbance at 517 nm. Ascorbic acid was used as a standard.

The following equation determined the percentage of inhibition:-

% inhibition = (Absorbance of control-Absorbance of test sample) × 100/Absorbance of control

The values of % inhibition at each concentration of the Camellia sinensis-mediated CuO nanoparticles were recorded and tabulated.

Antioxidant activity analysis by H_2_O_2_ assay

0.5 mL of 1 mM ferrous ammonium sulphate was added to each of 5 test tubes followed by 0.13 mL of 5 mM H_2_O_2_ and 3 mL of the Camellia sinensis-mediated CuO nanoparticles at varying concentrations of 10 µL, 20 µL, 30 µL, 40 µL and 50 µL. Each test tube was incubated in the dark for 5 mins at room temperature. After this, 3 mL of 1 mM 1,10-phenanthroline was added to each mixture and the test tubes were shaken to ensure uniform mixing. The reaction mixture was then incubated at room temperature for 10 mins. The absorbance of the reaction mixture was recorded at 510 nm. Gallic acid was used as a standard control drug. The amount of hydrogen peroxide scavenged was obtained from the following equation:-

% inhibition = (Absorbance of control-Absorbance of test sample) × 100/Absorbance of control

The values of % inhibition at each concentration of the Camellia sinensis-mediated CuO nanoparticles were recorded and tabulated.

Cytotoxicity analysis by zebrafish embryonic toxicology

The cytotoxicity of the nanoparticles was tested by using zebrafish embryos. The zebrafish embryos were treated with the Camellia sinensis-mediated CuO nanoparticles at different concentrations (0.5 μg/mL, 1 μg/mL, 2 μg/mL, 3 μg/mL, 4 μg/mL, and 5 μg/mL) during the post-fertilization period. The treatment was done in a 24-well plate where the wells were cut using a 9 mm sterile polystyrene tip. The hatching rate of the eggs, the viability rate of the embryos, and the morphology of the zebrafish were monitored every 24 hours throughout the experiment up to 96 hours post fertilization.

## Results

Scanning electron microscopy imaging

**Figure 4:**
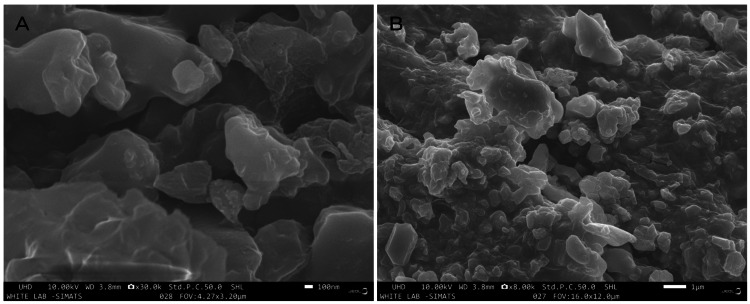
SEM analysis of Camellia sinensis mediated CuO nanoparticles at a focus of (A) 100 nm (B) 1 µm The Scanning Electron Microscopy (SEM) imaging shows the nanoscale size and crystalline morphology of the CuO nanoparticles.

The SEM images showed the crystalline morphology of the nanoparticles, which increased in cresting and troughing as the focus was zoomed in from the nanoscale to the microscale (Figure 4).

Energy dispersion X-ray (EDX) analysis

**Figure 5:**
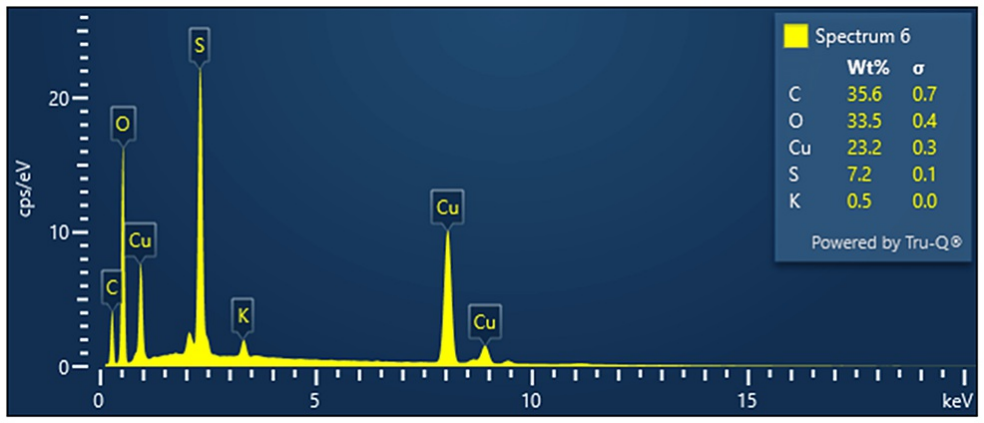
EDX analysis of Camellia sinensis-mediated CuO nanoparticles The EDX analysis showed a maximum number of peaks corresponding to elemental copper, confirming CuO nanoparticle synthesis from the Camellia sinensis extract.

The EDX analysis showed the highest number of peaks corresponding to the energy of elemental copper (Figure 5).

Antioxidant assay

**Figure 6:**
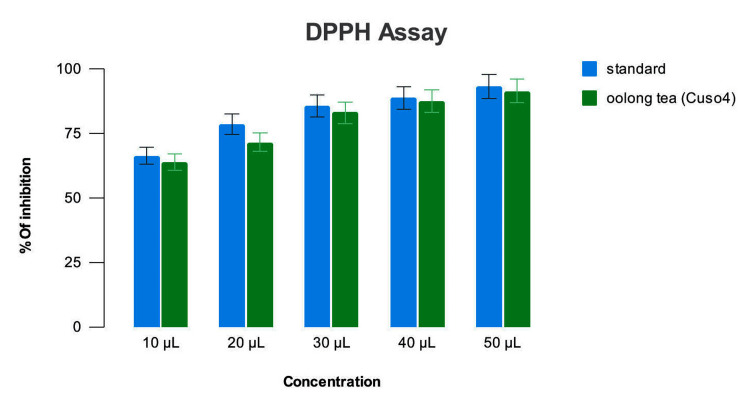
DPPH assay of Camellia sinensis-mediated CuO nanoparticles The antioxidant property analyzed using DPPH (2,2 - diphenyl - 1 - picryl hydrazyl) assay had evinced a comparable free radical scavenging activity of the nanoparticles to that of the standard (ascorbic acid).

The Camellia sinensis-mediated copper oxide nanoparticles on DPPH assay showed 63% inhibition at 10 µL concentration, 73% inhibition at 20 µL, 80% at 30 µL, 85% at 40 µL and 90% at 50 µL concentrations. The standard ascorbic acid showed 60% inhibition at 10 µL, 65% at 20 µL, 70% at 30 µL, 73% at 40 µL and 83% at 50 µL (Figure 6).

The antioxidant property of the CuNPs showed a proportionate increase with the increase in concentration compared to the standard.

**Figure 7:**
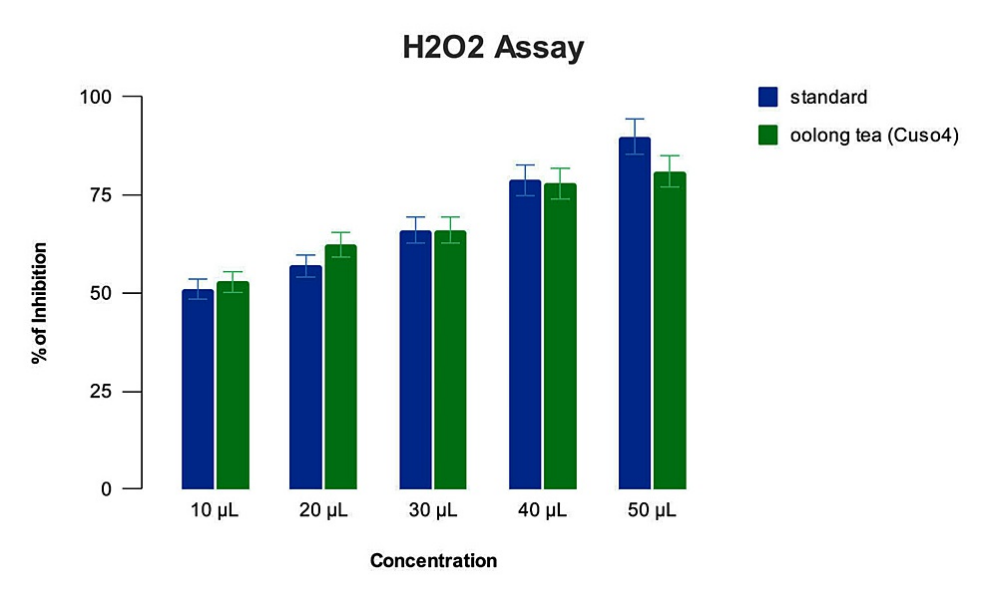
H2O2 assay of Camellia sinensis-mediated CuO nanoparticles The antioxidant activity measured by the H_2_O_2_ (hydrogen peroxide) assay has shown that at lower concentrations (below 40 µL), the copper oxide nanoparticles have higher free radical scavenging activity than the standard (ascorbic acid), while at higher concentrations (above 40 µL), the copper oxide nanoparticles have lower free radical scavenging activity than the standard (ascorbic acid).

The Camellia sinensis-mediated copper oxide nanoparticles on H_2_O_2_ assay showed 50% inhibition at 10 µL concentration, 65% at 20 µL, 68% at 30 µL, 75% at 40 µL and 80% at 50 µL concentrations. The standard control ascorbic acid showed 50% inhibition at 10 µL, 58% at 20 µL, 68% at 30 µL,75% at 40 µL and 90% at 50 µL concentrations (Figure 7).

Zebrafish embryonic toxicology

**Figure 8:**
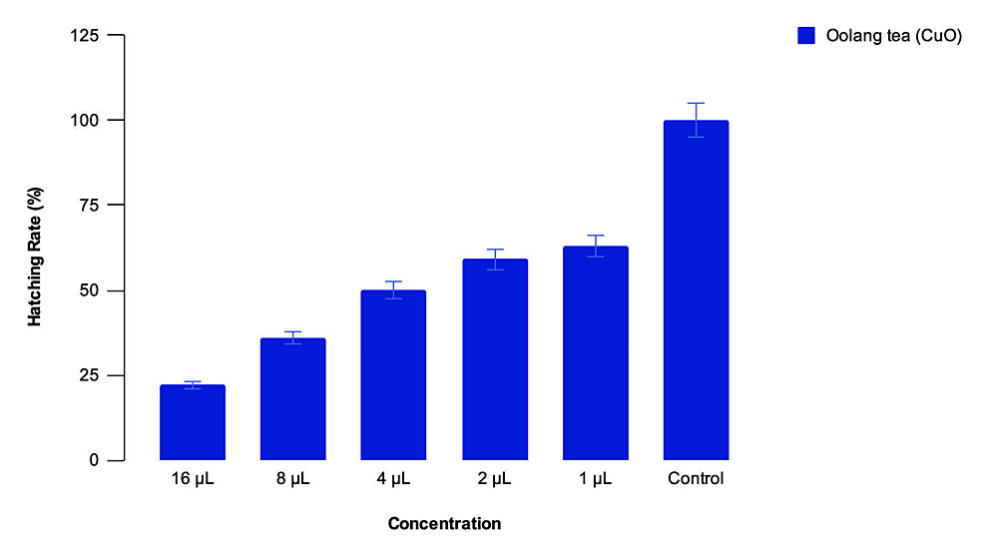
Hatching rate of zebrafish embryos against concentration of Camellia sinensis-mediated CuO nanoparticles The zebrafish embryo toxicology test has shown that the nanoparticles have achieved a 50-75% hatching rate at 1-4µL concentration, beyond which there is reduced hatching activity.

There was timely hatching of 25% of embryos at 16 µL concentration of nanoparticles, 38% at 8 µL, 50% at 4 µL, 60% at 2 µL and 65% at 1 µL concentrations, while there was 100% timely hatching in the blank control (Figure 8).

**Figure 9:**
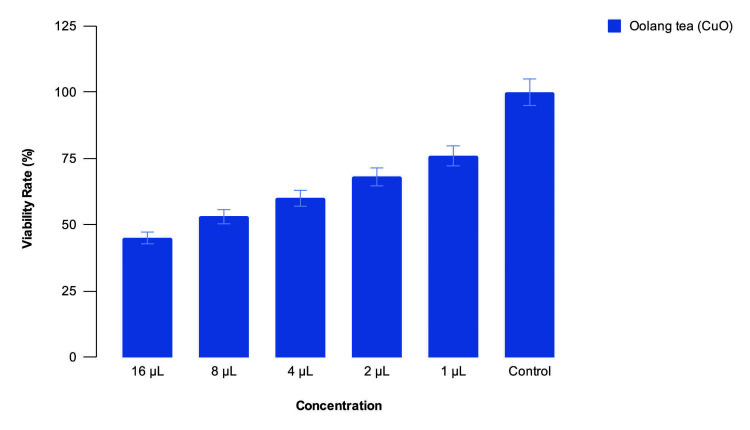
Viability rate of zebrafish embryos against concentration of Camellia sinensis-mediated CuO nanoparticles The zebrafish embryo toxicology test has shown 50-75% viability at the concentrations of 1-8µL of the nanoparticles, beyond which there is a decrease in viability rate.

There was a viability rate of 45% zebrafish embryos at 16 µL concentration of nanoparticles, 50% at 8 µL, 60% at 4 µL, 65% at 2 µL and 75% at 1 µL, while there was 100% embryo viability in the blank control (Figure 9).

**Figure 10:**
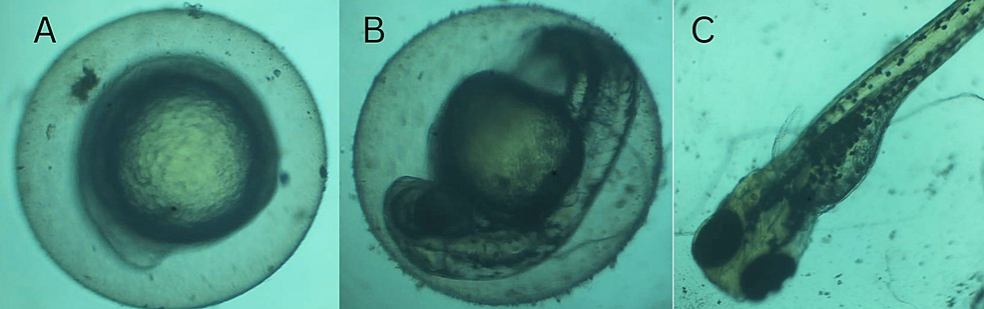
Zebrafish embryonic toxicology imaging of Camellia sinensis-mediated CuO nanoparticles A) Day 1 shows partially formed healthy zebrafish embryos inside the egg; B) Day 2 shows fully formed healthy zebrafish embryos inside the egg; C) Day 3 shows fully hatched healthy zebrafish

The morphology imaging showed the half-formed viable embryos inside the egg on day 1, the fully formed viable embryos inside the egg on day 2, and the hatched healthy zebrafish on day 3. There were no somatic deformities such as bent tail or bent spine and no evidence of oedema (Figure 10).

## Discussion

Scanning Electron Microscopy (SEM) imaging shows the size and shape of the Camellia sinensis-mediated CuO nanoparticles or oolong tea. In this study, we saw that the size of the nanoparticles ranged in nanometers. The nanoparticles had a highly crystalline morphology, with the cresting and troughing increasing the further zoomed in the focus was.

The Energy Dispersive X-ray (EDX) analysis shows the maximum number of peaks in the energy level corresponding to elemental copper. This is a confirmatory signal of the presence of copper-influenced nanoparticles in the test sample. Thus, we have confirmed that copper oxide nanoparticles were synthesized from the Camellia sinensis extract.

The 2,2 - diphenyl - 1 - picryl hydrazyl (DPPH) assay is a popular, quick, easy, and affordable approach for the measurement of the antioxidant properties of compounds. The assay includes free radicals used to assess the potential of substances for free-radical scavenging (FRS) or providing hydrogen. The technique is associated with eliminating stable free radical DPPH, which interacts with an odd electron to give a strong peak in the absorbance spectrum at 517 nm, resulting in a purple hue. As soon as the DPPH free radicals are combined with the hydrogen atom source, the lower state of diphenyl picryl hydrazine is formed, resulting in the loss of violet color, significantly affecting the lowering capacity [14].

This phenomenon of free radical elimination is utilized to assess the radical scavenging activity of compounds as a measure of their antioxidant property. The % inhibition value obtained shows the free radical's elimination amount, which shows the number of hydrogen atoms released by the test compound. In the case of the Camellia sinensis-mediated CuO nanoparticles, the percentage of inhibition was highest at 50µL concentration, as shown in the DPPH assay and the supplementary H_2_O_2_ assay. The results obtained were in line with those previously observed by [3,5,15,16].

Zebrafish or Danio rerio is a common vertebrate disease model used to study the effects of toxic chemicals or to analyze whether a newly synthesized drug or a newly discovered chemical compound can adversely affect humans [17]. The small size of the zebrafish, their development outside of the mother, and the low maintenance cost of the adult contribute to the zebrafish being a suitable experimental model. Experimenting with zebrafish embryonic systems can help identify the mechanisms of congenital disorders arising from genetic mutations. [18] Testing parameters commonly used for zebrafish include embryo survival rate, lethality, behavior, and organ deformity. Researchers have found that zebrafish exhibit a strong dose reaction to toxicity, making it a useful animal model for toxicity screening. [19]

There is timely hatching of the embryos at all concentrations below 4µL of nanoparticles. There is 80% cell viability at all concentrations below 8µL of nanoparticles. There are no somatic deformities, such as a bent tail or bent spine, and no evidence of oedema, which shows the non-cytotoxic nature of the nanoparticles. These results are in line with those obtained by [17] [18] and [20]. Since the nanoparticles are proven to be non-cytotoxic at low concentrations and a very good antioxidant, suitable compounds that they can be incorporated into can be identified by further research, and then they can be used in therapeutics and pharmaceuticals.

The limitations faced by this study is that there may be natural death of the zebrafish embryos happening during the course of the experiment. This may interfere with the results of the toxicology screening. Nanoparticle synthesis in itself presents a number of challenges, as enumerated by [21].

## Conclusions

In this study, copper oxide nanoparticles are successfully synthesized from *Camellia sinensis* or oolong tea using copper sulfate. From the DPPH and H_2_O_2_ assays, these nanoparticles are shown to have high antioxidant activity. The zebrafish embryonic toxicology assay shows that the synthesized nanoparticles have minimal cytotoxic activity at higher concentrations since there are no somatic deformities in the zebrafish embryos. Thus, it is confirmed that copper oxide nanoparticles can be used safely in therapeutics in combination with other suitable compounds as biocompatible antioxidants.
